# Acute postoperative pain in 23 procedures of gynaecological surgery analysed in a prospective open registry study on risk factors and consequences for the patient

**DOI:** 10.1038/s41598-021-01597-5

**Published:** 2021-11-12

**Authors:** Jorge Jiménez Cruz, Angela Kather, Kristin Nicolaus, Matthias Rengsberger, Anke R. Mothes, Ekkehard Schleussner, Winfried Meissner, Ingo B. Runnebaum

**Affiliations:** 1 Department of Gynecology and Reproductive Medicine, Jena University Hospital, Friedrich Schiller-University Jena, Am Klinikum 1, 07747 Jena, Germany; 2 Department of Obstetrics, Jena University Hospital, Friedrich Schiller-University Jena, Am Klinikum 1, 07747 Jena, Germany; 3 Department of Anaesthesiology and Intensive Care, Jena University Hospital, Friedrich-Schiller-University Jena, Am Klinikum 1, 07747 Jena, Germany; 4 Present Address: Department of Obstetrics and Perinatal Medicine, Bonn University Hospital, Sigmund Freud Street 25, 53127 Bonn, Germany; 5 Present Address: Department of Gynaecology, St. Georg Hospital Eisenach, Academic Teaching Hospital of University of Jena, Mühlhäuser Straße 94, 99817 Eisenach, Germany; 6 Present Address: SRH Wald-Klinikum Gera, Gera, Germany

**Keywords:** Pain management, Surgery

## Abstract

Effective perioperative pain management is essential for optimal patient recovery after surgery and reduces the risk of chronification. However, in clinical practice, perioperative analgesic treatment still needs to be improved and data availability for evidence-based procedure specific analgesic recommendations is insufficient. We aimed to identify procedures related with high pain scores, to evaluate the effect of higher pain intensity on patients and to define patient and intervention related risk factors for increased pain after standard gynaecological and obstetrical surgery. Therefore, we performed a prospective cross-sectional study based on the German registry for quality in postoperative pain (QUIPS). A cohort of 2508 patients receiving surgery between January 2011 and February 2016 in our tertiary referral centre (university departments of gynaecology and obstetrics, respectively) answered a validated pain questionnaire on the first postoperative day. Maximal pain intensity was measured by means of a 11-point numeric rating scale (NRS) and related to procedure, perioperative care as well as patient characteristics. The interventions with the highest reported pain scores were laparoscopic removal of ovarian cysts (NRS of 6.41 ± 2.12) and caesarean section (NRS of 6.98 ± 2.08). Factors associated with higher pain intensity were younger age (OR 1.75, 95% CI 1.65–1.99), chronic pain (OR 2.08, 95% CI 1.65–2.64) and surgery performed outside the regular day shift (OR 1.67, 95% CI 1.09–2.36). Shorter duration of surgery, peridural or local analgesic and preoperative sedation reduced postoperative pain. Patients reporting high pain scores (NRS ≥ 5) showed relevant impairment of daily activities and reduced satisfaction. Caesarean section and minimal invasive procedures were associated with the highest pain scores in the present ranking. Pain management of these procedures has to be reconsidered. Younger age, receiving surgery outside of the regular shifts, chronic pain and the surgical approach itself have a relevant influence on postoperative pain intensity. When reporting pain scores of 5 or more, patients were more likely to have perioperative complications like nausea or vomiting and to be impaired in mobilisation. Registry-based data are useful to identify patients, procedures and critical situations in daily clinical routine, which increase the risk for elevated post-intervention pain. Furthermore, it provides a database for evaluation of new pain management strategies.

## Introduction

Standard surgical procedures performed in gynaecology and obstetrics can be associated with severe postoperative pain^1^. Furthermore, pain management in female patients may be more complex compared to male patients. Female patients reported higher scores for acute pain^2^, had a higher risk of developing chronic pain^3,4^ and sometimes did not benefit from standard analgesic medication the same way as male patients did^5–7^. High pain intensity scores after surgery are associated with negative consequences such as decreased patient satisfaction, delayed rehabilitation, chronification of pain and increased morbidity and mortality^8–10^. Although procedure-specific strategies for reduction of postoperative pain have been established for surgeries such as laparoscopy for pelvic disease or caesarean section, gynaecological and obstetrical surgeries belong to the interventions with highest postoperative pain scores^11–13^. Therefore, surgical techniques as well as demographic factors increasing risk of significant postoperative pain need to be investigated in more detail in order to develop specific strategies for preventing and managing postoperative pain. Particularly, improvement of perioperative pain management with regard to reduction of opioid consumption is a contemporary issue^14,15^.

This study aimed to identify procedures associated with higher pain scores as well as perioperative and demographic risk factors for increased post-operative pain in women undergoing surgical procedures in gynaecology and obstetrics. Secondary aims were (1) Assessment of the relationship between increased pain scores and postoperative patient impairment, satisfaction and side effects of therapy. (2) Evaluation of the effect of healthcare management aspects on pain intensity, including timing of surgery and use of new analgesic treatment options.

## Methods

### Study design

This is a cross-sectional register-based study performed at the department of gynaecology and obstetrics in the Jena University Hospital, Germany, a tertiary referral centre for gynaecological surgery and obstetric medicine. All methods used in this study were performed in accordance with the relevant guidelines and regulations. Study approval was obtained from the responsible ethics committee. Before entering the study, all patients gave their written informed consent.

### Study period and pain registry

For this study, data from our centre collected for the “Quality Improvement in Postoperative Pain Treatment” project (German: QUIPS) between January 2011 and February 2016 were used. QUIPS (and its international counterpart PAIN OUT, www.pain-out.eu) is a multicentre initiative to evaluate intern quality of acute postoperative analgesic management in all surgical disciplines and to compare pain outcome parameters among participating hospitals through a benchmarking tool^16^. As defined for this project, data collection in an open registry is standardized and all participants receive a specific training and instruction before taking part in the project. With more than 639,000 records, the German QUIPS represents the most comprehensive database for acute pain worldwide.

### Patient selection

Patients were eligible if they met the following criteria: 18 years old and above; gave informed consent; answered the questionnaire on the first day after surgery, underwent an inpatient surgical procedure. Exclusion criteria were refusal of answering the questionnaire or participation in another clinical study.

### Data sampling

A specifically trained external research assistant visited all patients on first day (24–32 h) after surgery to collect demographic, clinical and outcome data. Patients were instructed to anonymously fill in a validated pain questionnaire assessing patient-reported outcomes (QUIPS questionnaire, https://www.quips-projekt.de/services/dateien). If a patient was unable to fill out the questionnaire by herself, the research assistant interviewed the patient. The questionnaire was developed and validated^17,18^ by the pain-unit of the Jena University Hospital and is divided into different domains addressing pain intensity, functional impairment, side effects of pain treatment, and global assessment of postoperative pain management by the patient (Table 1). Pain intensity was assessed by using an 11-point numeric rating scale (NRS) with 0 representing no pain, and 10 representing most severe pain. 11-point-NRS was also used for satisfaction with analgesic therapy being 0, completely unsatisfied and 10, completely satisfied.

**Table 1:** Overview of outcome measures on the questionnaire (translated from German).

Outcome measure	Scale
**Overview of outcome measures on the questionnaire**	
Pain on ambulation/stress	NRS 0–10*
Maximum pain intensity since surgery	NRS 0–10*
Minimum pain intensity since surgery	NRS 0–10*
Is pain interfering with your mobility or movement?	Yes/no
Are you experiencing pain when you cough or breathe deeply?	Yes/no
Were you woken up by pain last night?	Yes/no
Is pain interfering with your mood?	Yes/no
Have you felt very tired since your surgery?	Yes/no
Have you felt nausea since your surgery?	Yes/no
Have you vomited since your surgery?	Yes/no
Would you have liked to have received more pain medication?	Yes/no
How satisfied are you with your pain treatment since surgery?	NRS 0–10**

*Numeric Rating Scale (NRS) for pain: 0 = no pain, 10 = most intense pain imaginable.

**NRS for satisfaction: 0 = completely unsatisfied, 10 = completely satisfied.

Demographic and clinical parameters were documented at the same time. No member of the surgical or ward team was involved in pain data collection or patient questioning. To ensure standardised data collection, written guidelines and trainings were provided to study personnel. All data were collected and sent to a central web-based database as described for the QUIPS project^16^.

Data were continuously collected for the first year and in periods of 4–6 months in the following years.

### Analgetic strategies and surgical procedures

Within the study period, two new strategies for postoperative pain management were introduced into the department. Intraoperative use of local anaesthetics at the surgical site was implemented sequentially in laparoscopic surgery^12^, Caesarean sections^19^ and breast surgery^20^. The entire surgical team was instructed and trained in the application technique to guarantee uniformity of the procedure. Furthermore, the prophylactic use of orally administered oxycodone 10 mg/naloxone 5 mg was included for breast surgery and laparoscopic myomectomy. This therapy was initiated 12 h prior to surgery, administered every 12 h and continued until the second day after surgery.

A standard procedure-specific, anaesthetic technique was employed in all procedures as defined in Standard Operating Procedures (SOPs) of the department without changes within the study period, as described in detail in the supplemental material. Also, surgical procedures were developed following specific internal SOPs. Rationale for special surgical approaches is given in the supplemental information.

### Statistical analysis

In order not to lose statistical accuracy, procedures performed in less than 10 patients during the study period were excluded from the analysis. Categorical variables were analysed using chi-squared or Fischer’s exact test as appropriate. For the analysis of risk factors for increased pain, a multivariate model was created and analysed using a binary logistic regression. For this regression, increased pain was defined if maximal pain intensity score was 5 or more. This cut-point^21^ value defines moderate to severe pain, and is known to correlate with the negative impact of pain in physical and emotional aspects of patient care^22–24^. Only variables which showed a significant relationship with increased pain in the chi-squared test were included in that multivariate analysis. After identifying relevant factors influencing pain scores in the multivariate model, pain intensity scores were recalculated adjusting for those factors using the regression equation. Specifically, adjustment was performed for the variables age, surgical approach, use of innovative pain strategies, chronic pain patient and timepoint of beginning of surgery. These adjusted values were used for the comparative ranking of pain intensity.

To asses relationship between duration of surgery and maximal pain intensity Spearman’s correlation was used since both variables are linear. For the variable timepoint of beginn of surgery, a binary value was defined by using the cut-off time of 4:30 PM, because this was the time scheduled surgery ends in our centre. Statistical significance was considered if *p* values were < 0.05. Statistical analysis was performed using SPSS version 20.0 (SPSS Inc. Chicago, IL).

### Ethics approval

This research was covered by the study approval for the German registry for Quality of management on postoperative pain (QUIPS) of the Ethics Commission of the Jena University Hospital, Germany. Registry-Number 2722-12/09 from the 8th of December 2009.

### Paper presentation information

Study outline presented by Ingo B. Runnebaum at the 22nd Annual Congress of the European Society of Gynaecological Endoscopy, 16–19 October 2013, Berlin. Concept and preliminary data of the study were orally presented at the 61st Congress of the German Society for Gynaecology and Obstetrics (DGGG), Oct. 19.-22., 2016, Stuttgart, Germany.

## Results

### Patients and surgical procedure ranking

**Figure 1:**
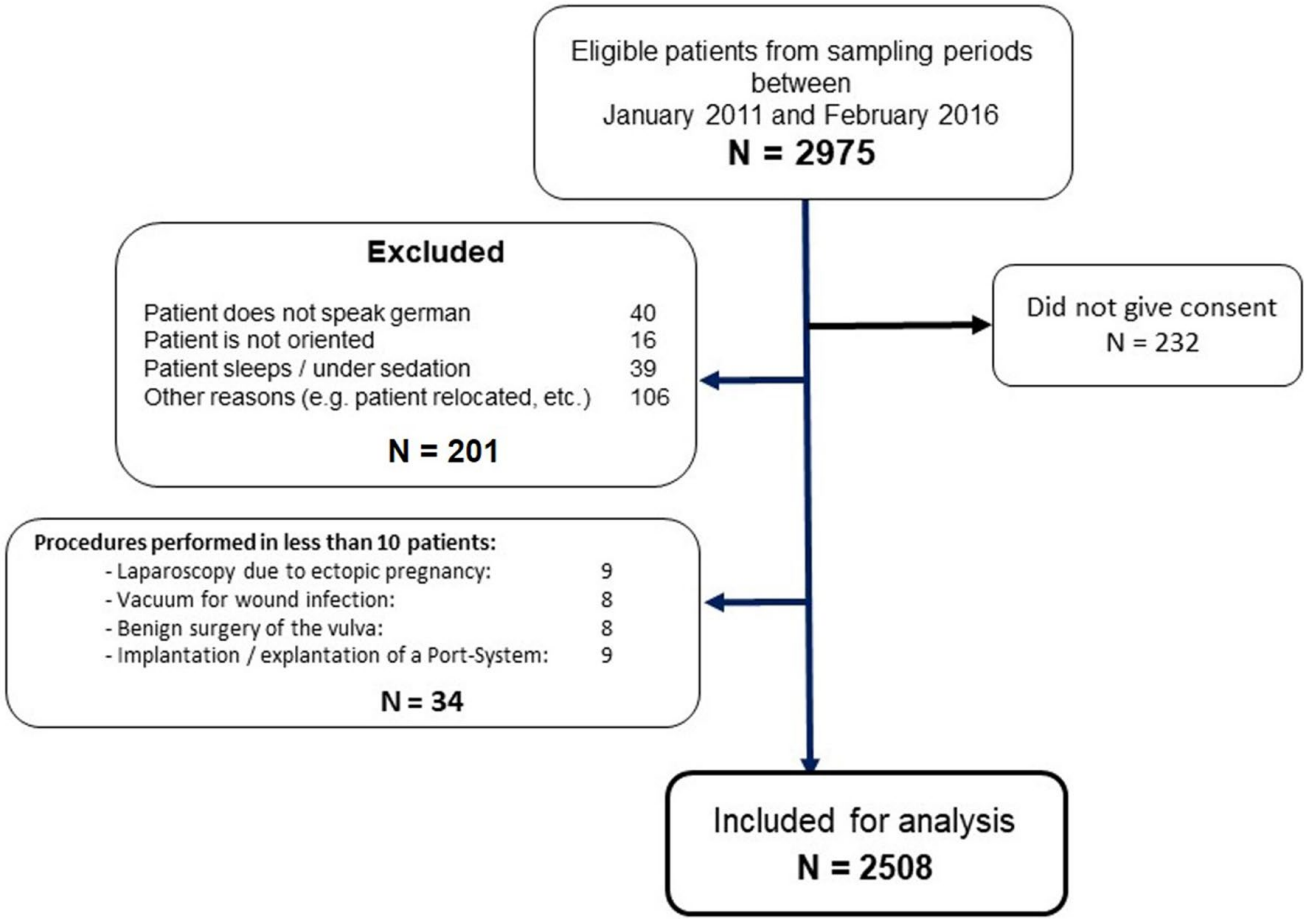
Patient inclusion process.

**Figure 2:**
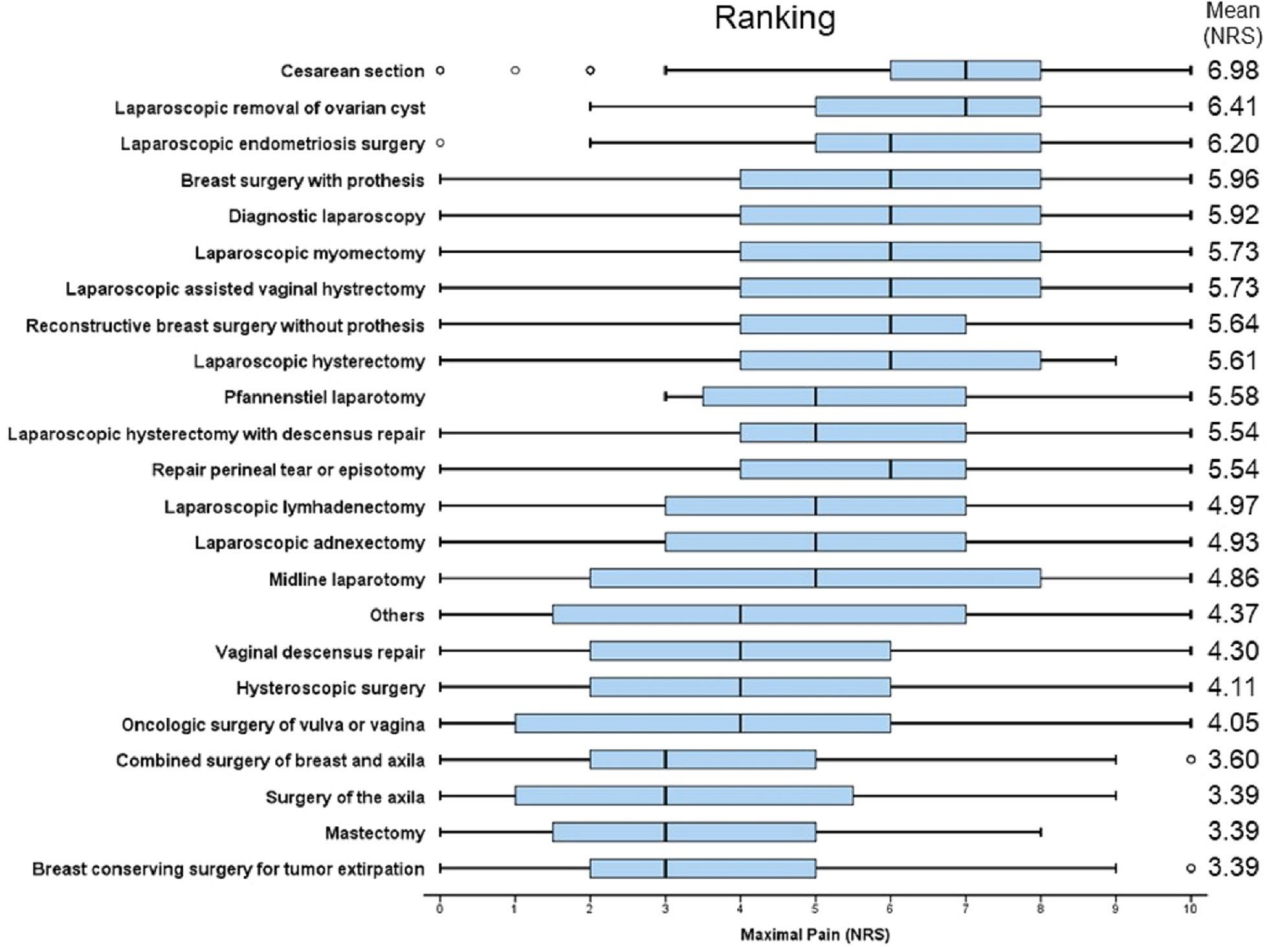
Ranking of postoperative pain depending on type of surgery. Postoperative pain scores 24-32 h after surgical procedures. Horizontal box plots indicate worst pain since surgery on 11-point numeric rating scale (NRS). Box edges indicate 25th and 75th percentiles. Whiskers indicate 5th and 95th percentiles. Procedures ranked in descending order of median pain severity. Mean scores (also shown) were used to rank surgical groups with identical median NRS scores. NRS scores were adjusted for age, surgical approach, use of innovative pain strategies, chronic pain patient and time of beginning of surgery using the regression equation.

Between January 2011 and February 2016, 2975 patients were eligible to be included in the study sample. 232 patients refused the questionnaire, 201 patients did not complete it (reasons given in Fig. 1) and 34 were excluded from data analysis due to not achieving the minimal amount of 10 patients per procedure. These were 9 patients with ectopic pregnancy, 8 patients with wound-debridement or vacuum therapy, 8 patients being operated on benign changes of the vulva and 9 patients after implantation or explantation of a PORT-system. A total of 2508 patients were prospectively included and analysed. Demographic data is presented in Table 2. Analysis of pain intensity showed that laparoscopy for ovarian cysts or endometriosis and Caesarean section were associated with the highest pain intensity scores in our hospital (Fig. 2: Complete ranking and pain scores adjusted for age, surgical approach, use of innovative pain strategies, chronic pain patient and time of beginning of surgery according to multivariate regression analysis.).

**Table 2:** Patient characteristics and surgical procedures.

	n	% (within groups)
**Patient with chronic pain**		
No	1970	78.5
Yes	514	20.5
Unknown	24	1.0
Total	2508	100.0
**Age (years)**		
18–30	520	20.7
31–40	512	20.4
41–50	448	17.9
51–60	395	15.7
61–70	339	13.5
Older than 71	269	10.8
n.a	25	1.0
Total	2508	100.0
ASA-Status*		
unknown	344	13.7
ASA I	611	24.4
ASA II	1296	51.7
ASA III	257	10.2
Total	2508	100.0
**Type of surgery depending on surgical approach**		
**Caesarean section**		
Caesarean section	409	16.3
**Laparoscopic approach (n = 609, 24.2%)**		
Laparoscopic hysterectomy	36	1.4
Laparoscopic lymphadenectomy	71	2.8
Diagnostic laparoscopy	128	5.1
Laparoscopic endometriosis surgery	60	2.4
Laparoscopic removal of ovarian cyst	126	5.0
Laparoscopic adnexectomy	117	4.7
Laparoscopic myomectomy	71	2.8
**Combined laparoscopic and vaginal approach (n = 255, 10.1%)**		
Laparoscopic assisted vaginal hysterectomy	144	5.7
Laparoscopic hysterectomy and descensus repair	111	4.4
**Vaginal surgery (n = 159, 6.2%)**		
Vaginal descensus repair	137	5.5
Oncologic surgery of vulva or vagina	22	0.9
**Perineal repair**		
Repair of perineal injury after delivery	225	9.0
**Breast surgery (n = 668, 26.7%)**		
Reconstructive breast surgery	47	1.9
Breast preserving tumor extirpation	203	8.1
Mastectomy	24	1.0
Breast surgery with prosthesis	51	2.0
Surgery of the axilla	28	1.1
Combined surgery of breast and axilla	315	12.6
**Laparotomy (n = 76, 3.1%)**		
Midline laparotomy for tumor removal	64	2.6
Pfannenstiel laparotomy for tumor removal	12	0.5
**Other procedures (n = 10, 4.3%)**		
Other unspecified procedures^#^	60	2.4
Hysteroscopic surgery	47	1.9
Total	2508	100.0

*ASA-Status American society Anaesthesiology, *ASA 1* no organic pathology, *ASA 2* moderate but definite systemic disturbance, *ASA 3* Severe systemic disturbance from any cause, *ASA 4* Extreme systemic disorders which have already become an eminent threat to life regardless, *ASA 5* Moribund patient with little chance of surviving, *ASA 6* Brain-dead organ donor.

^#^In this group were included minor surgeries performed in at least 10 patients but not classifiable in other groups, for example because two different procedures were performed at the same time. These were 25 procedures combining hysteroscopy and laparoscopy, 13 patients with combined vulva and hysteroscopic surgery, 10 procedures for wound revision and 12 procedures involving removal of cutaneous or subcutaneous findings).

### Demographical risk factors

**Figure 3:**
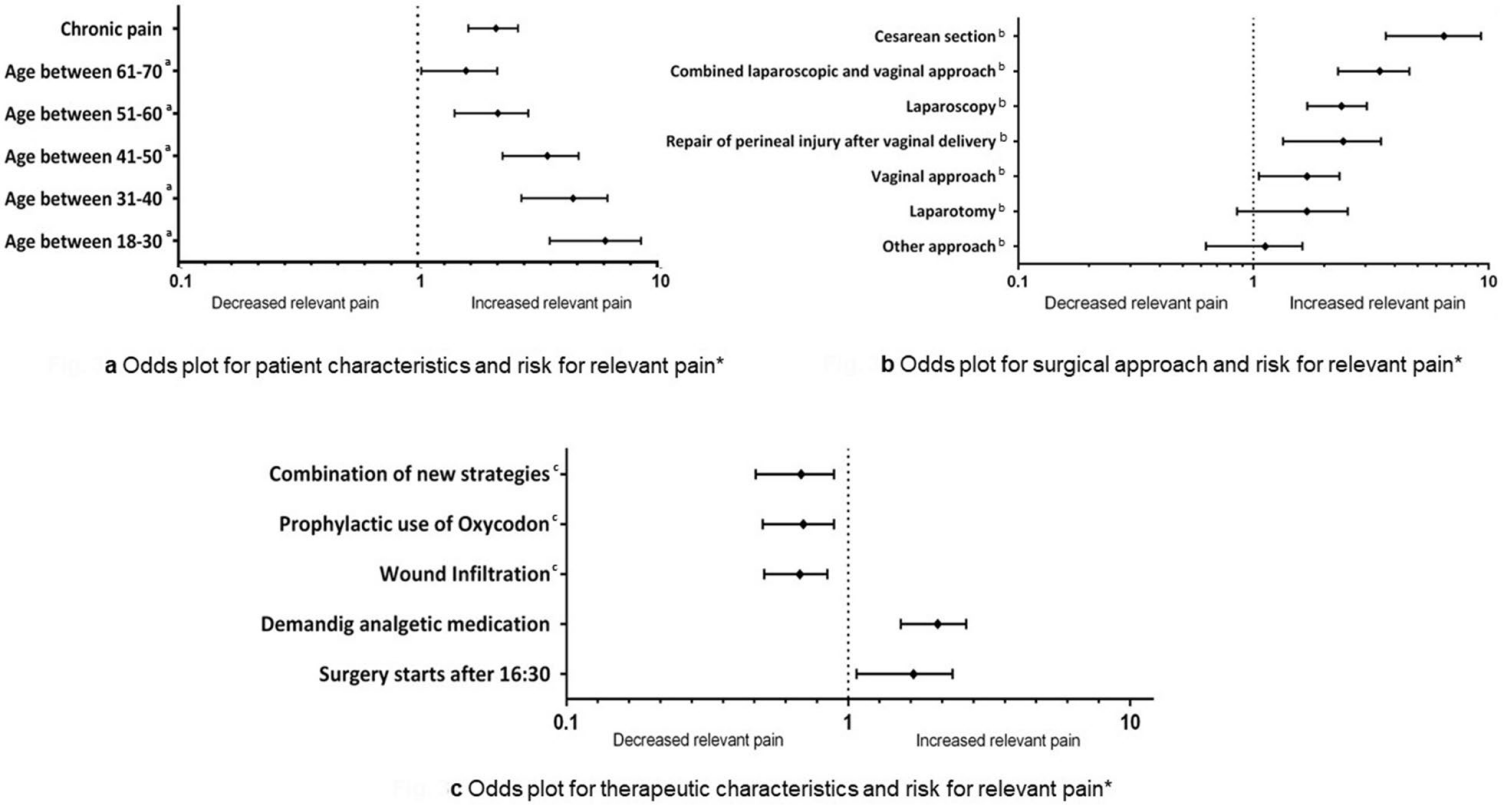
Association between risk factors and risk of increased pain. Binary logistic regression analysis adjusted by age, chronic pain, surgical approach, time point of surgery, use of wound infiltration, prophylactic oxycodone, opioids and epidural catheter. Reference variables: (**a**) age > 71 years; (**b**) breast surgery; (**c**) standard care.

Patients at a higher age reported significant lower pain intensity scores than younger patients. Patients over 71 years old reported the lowest pain scores and were used as the reference group for the regression (Fig. 3a). When using a cut-off of 50 years or younger, more patients showed increased pain score (NRS ≥ 5) compared to patients of 50 years and above (73.9% vs 42.2% respectively, OR 1.75 with a 95% CI 1.65–1.99, p < 0.001).

Patients receiving treatment for chronic pain prior to surgery (independently of location of chronic pain) were more likely to report higher pain scores (OR 2.08, 95% CI 1.65–2.64, p < 0.001).

### Surgical risk factors: type and timing of surgery

Surgeries were classified into seven groups depending on surgical area and surgical approach (Table 2): laparoscopic approach, caesarean section, breast surgery, laparotomy, vaginal surgeries, perineal repair of injury after delivery, combined laparoscopic and vaginal approach. Breast surgery was identified to be the type of surgery associated with the lowest pain scores, and was used as a comparator for the other types of surgery. In the binary logistic regression patients receiving laparoscopic or combined vaginal and laparoscopic surgery or caesarean section showed to be at higher risk of developing increased postoperative pain (ORs adjusted by age, chronic pain, surgical approach, time point of surgery, use of wound infiltration, prophylactic oxycodone, opioids and epidural catheter: 2.30, 95% CI 1.73–3.07 for laparoscopy; 3.32, 95% CI 2.36–4.66 for combined laparoscopy and vaginal approach; 6.06, 95% CI 3.88–9.46 for caesarean section) (Fig. 3c).

Comparison of elective and intra-partum caesarean section showed no significant difference (Mann–Whitney-U-Test) regarding pain on ambulation/stress (p = 0.888), maximum pain intensity (p = 0.557), minimum pain intensity (p = 0.394) and satisfaction with pain treatment (p = 0.672), respectively.

Performing subgroup analysis, we found no increased risk for elevated pain associated with laparoscopic endometriosis surgery compared to laparoscopic interventions in total (n = 60/871, OR = 1.67, 95% CI 0.89–3.15, p = 0.105). Compared to the standard laparoscopy/laparotomy, need for adhesiolysis (n = 271/935, OR = 0.91, 95% CI 0.67–1.22, p = 0.503) or need for uretherolysis (n = 27/935, OR = 1.35, 95% CI 0.56–3.23, p = 0.494) did not increase the risk for higher postoperative pain.

Regarding the duration of surgery, we found a significant correlation between longer surgeries and higher pain intensities. Spearman´s correlation index demonstrated a weak correlation (0.16, p < 0.001). Interventions on patients who reported elevated pain levels lasted longer than those on patients with lower pain: 127.8 min (SD ± 3.3) vs 104.4 min (SD ± 3.6) (p < 0.001).

Surgery started during late shifts (after 4:30 p.m. in our centre) was also associated with higher postoperative pain after adjustment for age, kind of surgery and chronic pain (Fig. 3b). 437 records from not scheduled surgeries were registered. These interventions were 175 caesarean sections (40%), 148 perineal repair after delivery (33.9%), 78 laparoscopic surgeries (17.8%), 18 breast surgeries (4.1%) and 4.2% other surgeries being performed in less than 5 people each (wound debridement, marsupialisation or hysteroscopy). If patients were operated during regular day shifts, 57.7% reported elevated pain levels. Starting surgery on night shift was related not only with higher pain scores, but also more patients (77.9%) reported increased pain (unadjusted OR 2.59, adjusted OR 1.67; p < 0.05). Patients operated in late-shifts reported also lower scores of satisfaction with pain management (7.3 ± 2.2 vs 7.7 ± 2.2, p < 0.001) and would have desired more analgesics within the first 24 h after surgery (19.4% vs 10.9%, p < 0.001). These patients also received significant less opioids (75.5% vs 89.4%, p < 0.001). For patients operated outside regular shifts documentation for pain scores could be found only in 66% of them. This is significant less than the documentation rate of 98% for patients on day-shifts (p < 0.001). Also, premedication rates with hypnotics were different in this group. Since caesarean sections and perineal repair after delivery do not receive these drugs we performed a comparison of this fact only for the other procedures resulting that patients being operated after 4:30 pm received significant less midazolam than scheduled surgeries (92.9% vs 98.2% respectively, p = 0.006).

### Analgesic management

Patients who requested on demand analgesic therapy also reported higher pain scores (Fig. 3b).

Epidural catheters for postoperative analgesia were used in 101 procedures. These were laparotomies in 23 cases (22.8%), laparoscopies in 16 cases (15.8%), 50 caesarean sections (49.5%), 7 surgeries of the vulva (6.9%) and 5 breast surgeries (5%). Use of epidural catheters for postoperative analgesia were related with lower pain intensity (33.3% vs 56.1%, p = 0.002). Also premedication showed to have a significant influence on reporting higher pain levels (53.2% vs 60.8% p < 0.001).

As described above, two novel strategies were implemented during the study period for reducing postoperative pain: Prophylactic use of Oxycodone 10 mg/Naloxone 5 mg and perioperative local infiltration of Ropivacaine at the surgical site. Within the study period 1705 (68%) patients received at least one of those interventions. Local infiltration alone was applied in 984 (39.2%) patients, prophylactic oxycodone alone was used in 375 (15%) patients and the combination of both was implemented in 346 (13.8%) procedures. Compared with standard therapies, both strategies combined but also independently from each other were found to have a protective effect on increased pain after surgery. The OR for prophylactic oxycodone was 0.67 (95% CI 0.51–0.90, p < 0.01) and for local anaesthetics was 0.66 (95% CI 0.50–0.85, p < 0.01), and the OR for the combined use of both strategies was 0.66 (p < 0.01) (Fig. 3b).

### Consequences of reporting high pain scores

**Figure 4:**
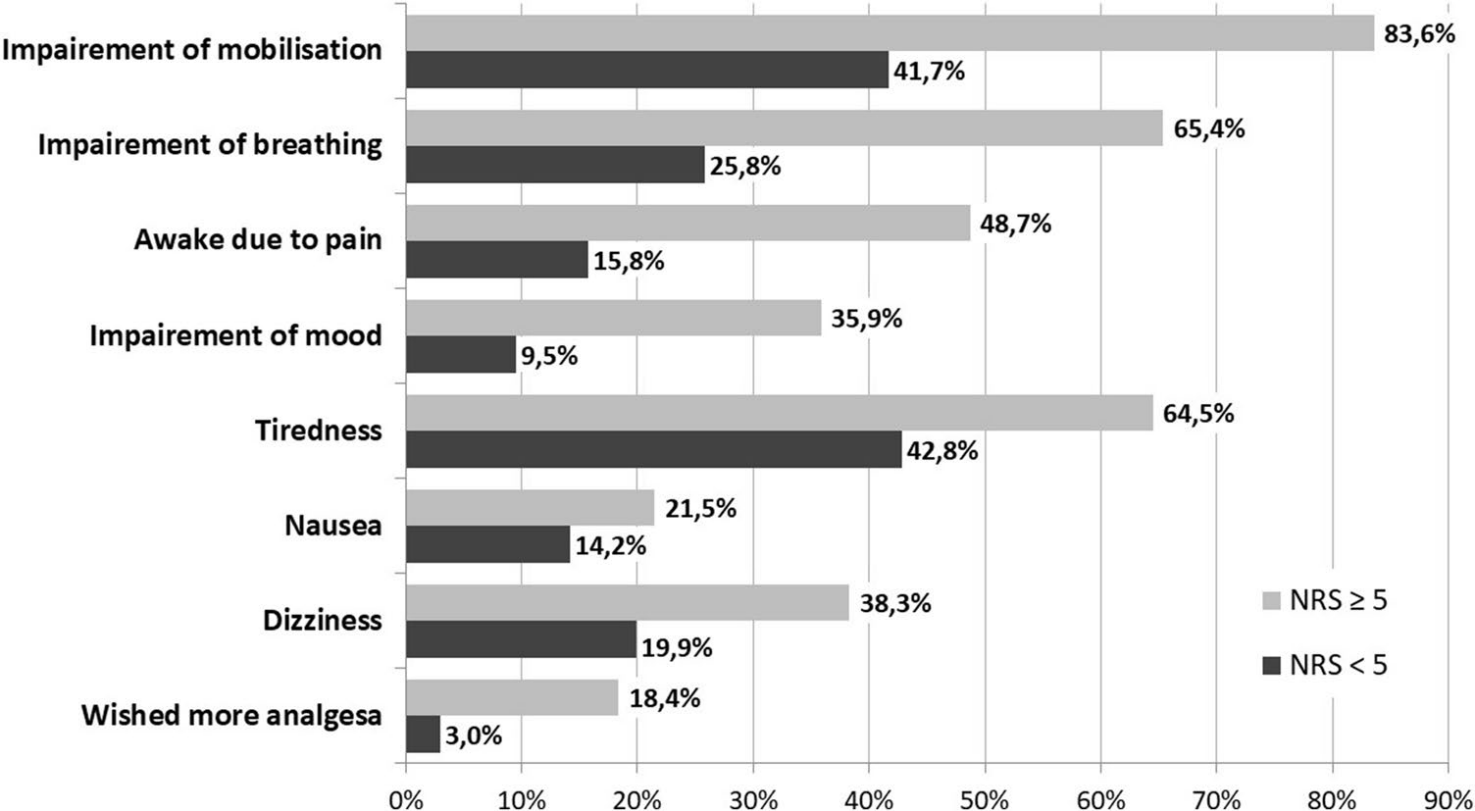
Comparison of side effects and impairment depending on pain score. Percentage of patients reporting side effects. Patients with high pain scores on 11-point numeric rating scale (NRS ≥ 5) on first postoperative day compared with patients showing low pain intensity (NRS < 5). Chi-Square-Test indicates a statistically significant difference for each comparison. (p < 0.05).

A remarkable number of patients (n = 1528, 61.2%) reported maximal pain scores of five or more in the NRS (increased pain). As shown on Fig. 4, these women were significantly more impaired in daily aspects and complained more often about side effects such as nausea and exhaustion, than patients with pain scores lower than 5 (p < 0.05 for all parameters). Reporting increased pain was also associated with the wish for more analgesics (Fig. 4). Satisfaction with pain management was significantly higher in the group with lower postoperative pain (NRS 8.2 ± 2.2 vs. 7.3 ± 2.2 p < 0.001).

## Discussion

To our knowledge, this is the largest prospective cross-sectional study to date comparing standardised pain intensity scores subsequent to a wide range of obstetric and gynaecologic surgeries, in search of risk factors for postoperative pain. This approach aimed to demonstrate the painfulness of routine interventions and provides a relation between painfulness, the treatment provided and patient characteristics.

Results of this study show that some minimally invasive laparoscopic procedures and caesarean section were associated with patients reporting higher pain scores. The results are consistent with the literature for caesarean sections^1^. Concerns about pharmaceutical analgesic therapy with respect to breast feeding and anxiety in the early postoperative time with respect to baby care have been discussed as factors contributing to high pain intensity^25^.

Advantages of laparoscopic surgery related to postoperative pain and postoperative recovery have been confirmed by well-designed large randomised studies^26–29^. The results of our study do not show this benefit with regard to pain for the first 24 h after surgery for our centre. This difference may be explained by different standards of care for these surgeries as well as patient characteristics. Patients after radical surgeries such as lymphadenectomy including radical hysterectomy or other surgeries with extensive tissue trauma received postoperative regional analgesia and routine visits by the acute pain service (APS), regularly optimising pain therapy. Patients with minor and less invasive procedures were expected to experience less pain, and often were excluded from advanced pain management by APS. Furthermore, patients undergoing laparoscopic surgeries for ovarian cysts or endometriosis frequently were younger than patients treated with hysterectomy or pelvic organ prolapse surgery. As a result of our study, younger age was an independent risk factor for developing higher pain intensity. This is consistent with evidence from other studies and other surgeries^30–33^.

In this study, patients with chronic pain reported significantly higher pain scores. This has been a well-defined risk factor in previous studies^33,34^.

An additional risk factor for higher pain was time of day when starting surgery. Further exploring the influence of time of day, we analysed patients operated in late-shifts. They reported lower scores of satisfaction with pain management and would have desired more analgesics after surgery. Routine documentation of pain scores by nursing team may have played a relevant role here, since documentation rate was more than 30% lower in the night shift. This seems relevant since patients with reduced pain documentation showed less opioid consumption. An explanation here may be the reduced nurse-patient ratio at night resulting on a less attentive patient care. Furthermore, kind of surgery, as well as pre- and postoperative medication could be less accurate at this time. Surgeries on night shift were mainly caesarean sections, laparoscopic cyst extirpations or extra uterine pregnancies. We observed a significant amount of surgeries not receiving hypnotics for premedication, also after excluding caesarean sections. Therefore, the whole process of analgesic management on night shifts needs to be reviewed.

Our results demonstrate the efficacy of new opioid-sparing analgesic treatment strategies such as prophylactic oxycodone and intraoperative wound infiltration with long-acting local anaesthetics.

Furthermore, data from our study are in accordance with earlier reports, which showed a clear correlation between higher pain scores and negative influence on postoperative recovery^9,35–38^. Patients with high pain intensity (NRS ≥ 5) reported increased side effects such as nausea and impairment of daily activities and were less satisfied with pain management. (Fig. 4). We used for this study a cut-off point for increased pain of 5 on the 11-point NRS, which is broadly accepted^23,24,39^.

Small-scale randomised studies or studies performed under controlled conditions were often unsuccessful to create practice-changing evidence to daily hospital environment^40,41^. QUIPS (www.quips-projekt.de) and its international version PAIN OUT (www.pain-out.eu) are simple to use tools open to any institution to monitor quality of care in pain management, particularly during the first 24 h, using well defined standardised patient related outcomes and combining this information with data coming from routine daily patient care and demographical data^42^. More than 200 hospitals already participate in this benchmark program. Patients and hospitals can benefit from such a benchmark registry visualizing one’s own improvements of routine postsurgical pain management.

After evaluating the findings of our study, the entire staff of our surgery unit has been informed. The surgeons of our unit were convinced of the benefit of the studied protocol and voted for a strict adherence to the tested pain management protocols. In daily routine the here published protocol has consistently been practiced since. This comprises situations, in which deviation from standard practice had been more likely (emergency surgery, operations performed outside the regular day shift).

Furthermore, local wound infiltration with Ropivacaine was implemented in the standard anaesthetic protocol for all surgical interventions. In perineal repair surgery, local anaesthetics with short duration of action (Lidocain) was replaced with Ropivacain with longer action.

As to limitations of this study, data have been collected in a single centre making it difficult to exclude bias due to centre related singularities. Data were collected discontinuously over 5 years between 2011 and 2016. Changes in perioperative pain management in our centre within this time period have been evaluated in the present study, so influence of other factors appears unlikely but cannot be excluded. Data were not presented directly following data acquisition, but after stable implementation into all clinical routine situations.

In order to reduce selection bias, a high number of patients were enrolled. The study centre is a high-volume reference centre for gynaecological benign and oncological surgery (by German standards), so no significant fluctuations on reference population were expected. Authors tried to reduce potential performance and measurement bias by excluding any member of the surgical or ward team from data collection or data management. External assessors were especially trained in a defined program for this task. Influence of other factors, which could act as confounders, such as grade of anxiety, level of education or social background, were not aim of this study and have not been evaluated.

Minimally invasive surgeries reported unexpected higher pain intensity compared with other gynaecologic procedures. As an internal control, Caesarean section was expectedly associated with highest postoperative pain scores of the spectrum of surgeries analysed. Younger patients, patients with the diagnosis of chronic pain and patients being operated outside of regular day shifts were at risk of developing increased pain. These patient groups were more likely to be handicapped due to pain and should be identified prior to surgery. The benchmark registry QUIPS/PAIN OUT enabled us to define risk criteria for postsurgical pain in order to improve analgesic management in gynaecologic patients. Specific types of surgery will need specific techniques to further tailor postoperative opioid-sparing pain-preventive measures, which should be evaluated in another prospective benchmark analysis or a randomized-controlled study.

### Supplementary Information

Supplementary Information.
