# Supplementary material for: Acute postoperative pain in 23 procedures of gynaecological surgery analysed in a prospective open registry study on risk factors and consequences for the patient
Source: Sci Rep. 2021 Nov 12;11:22148. doi: 10.1038/s41598-021-01597-5 (PMC8590005; doi:10.1038/s41598-021-01597-5)
Supplement: Supplementary file 1 — Supplementary Information. [file 41598_2021_1597_MOESM1_ESM.docx]

Acute postoperative pain in 23 procedures of gynaecological surgery analysed in a prospective open registry study on risk factors and consequences for the patient

*Jorge Jiménez Cruz, Angela Kather, Kristin Nicolaus, Matthias Rengsberger, Anke R. Mothes, Ekkehard Schleussner, Winfried Meissner, Ingo B. Runnebaum, MD*

**Supplemental Material:**

1) Standard procedure specific anaesthetic protocol

Patients included for scheduled surgeries were evaluated by an anaesthesiologist and a gynaecologist at least 24h prior to surgery and provided informed consent for both anaesthesia and surgical procedure. Possible risk factors for complications within anaesthesia, ASA-Status and risk for postoperative nausea and vomiting (PONV) was assessed and prophylactic use of antiemetic drugs was prescribed as necessary. For premedication midazolam orally was used for gynecologic procedures. Sodium citrate was used for cesarean section for reducing aspiration risk in case an emergency intubation was needed.

All laparotomies, laparoscopies and breast surgeries were performed using general anesthesia. For those procedures Propofol and Sufentanyl was used to induce anesthesia. Rocuronium was given to facilitate intubation and controlled ventilation. Anesthesia was maintained using sevoflurane. If patients were classified as at risk for PONV antiemetic drugs like ondansetron and metoclopramide were applied as described in SOPs. During the postoperative period, all patients received standardised analgesic medication: 1 g metamizole (dipyrone) every 6 hours and patient or nurse controlled intravenous 1.5 to 3 mg piritramide bolus on demand. If more than 30 mg piritramide in 24 hours was required, the patient was evaluated by a pain specialist and analgesic medication was individually adapted. If required, acetaminophen, ibuprofen or other opioids (only pethidine or Tilidine) could additionally be administered on demand depending on medical assessment. For extensive laparotomy and oncological procedures, postoperative epidural-analgesia was provided for all patients for at least 24 hours using ropivacaine.

Scheduled primary caesarean sections were performed under spinal anaesthesia using a combination of Sufentanyl and bupivacaine. Secondary caesarean sections were performed under general, spinal or epidural anaesthesia depending on emergency setting.

2) Rationale for special surgical approaches

Cesarean sections were performed by the modified Misgav Ladach method using Pfannenstiel incision.
In prolapse surgery with concurrent hysterectomy, laparoscopically assisted vaginal hysterectomy (LAVH) was introduced as the standard procedure in our department in 2005 to achieve opportunistic salpingectomy or prophylactic bilateral salpingo-oophorectomy in all cases with a minimum of intra- and postoperative complications^1^. In the majority of cases presenting Level I defect (pelvic floor defect level according to DeLancey), sacrospinous ligament fixation was performed. Concurrent perineorrhaphy was performed as a standard procedure for dehiscent introitus in all patients undergoing vaginal reconstructive prolapse surgery resulting in improved quality of life and subjective cure (manuscript in preparation).

**References:**

1. Mothes AR, Schlachetzki A, Nicolaus K, Vorwergk J, Lehmann T, Radosa MP, et al. LAVH superior to TVH when concomitant salpingo-oophorectomy is intended in prolapse hysterectomy: a comparative cohort study. Arch Gynecol Obstet. 2018;298(6):1131-7.
